# From ballooning to normal voiding: functional and cosmetic outcomes after congenital megaprepuce repair (a case report)

**DOI:** 10.11604/pamj.2026.53.35.50489

**Published:** 2026-01-27

**Authors:** Muhammad Asykar Palinrungi, Harry Achsan Chaerul, Muhammad Fakhri, Dedy Kurniawan

**Affiliations:** 1 Department of Urology, Faculty of Medicine, Hasanuddin University, Makassar, Indonesia,; 2 Department of Urology, Faculty of Medicine, Brawijaya University, Malang, Indonesia,; 3 Faculty of Medicine, Muslim University of Indonesia, Makassar, Indonesia

**Keywords:** Congenital megaprepuce, preputial ballooning, pediatric urology, case report

## Abstract

Congenital megaprepuce (CMP) is a rare congenital disorder characterized by excess mucosa accompanied by a partial thickening of the ventral prepuce. This abnormality causes complete penis coverage without any additional penile abnormalities, leading to ballooning of the prepuce due to urinary retention, which is the primary complaint of patients. We report a 4-year-old boy who came to the urology clinic with complaint of difficult micturition, pain, and ballooning of the prepuce during urination. The ballooning was from the accumulation of urine in the preputium. It happened since the patient was an infant. The patient sometimes felt a fever every single month but was getting better after taking medication. Local examination of genitalia showed the penis appeared buried beneath a large amount of skin of preputium, which was excessively redundant and also not circumcised yet. During micturition, urine collects within this sac, leading to ballooning of the prepuce, and urine dribbling through the small prepuce orifice. The patient was planning for surgical intervention to release the glans penis with circumcision and reconstruction of the penis. Congenital megaprepuce (CMP) needs to be differentiated from other penile anomalies such as buried penis and webbed penis. Early recognition and surgical intervention are essential to prevent complications and achieve satisfactory cosmetic outcomes for the circumcised penis.

## Introduction

Congenital megaprepuce (CMP) is a rare congenital disorder characterized by excess mucosa accompanied by a partial thickening of the ventral prepuce [1]. This abnormality causes complete penis coverage without any additional penile abnormalities, leading to ballooning of the prepuce due to urinary retention, which is the primary complaint of patients [2]. Congenital megaprepuce was first described in a case report by O'Brien *et al*. in 1994, with the patient presenting penile swelling and an inability to urinate spontaneously for eight months [3]. Congenital megaprepuce needs to be understood and differentiated from buried penis due to its frequent association with urinary tract infections, aesthetic abnormalities, and treatment differences from conventional phimosis [4]. Although CMP requires surgical treatment, it is different from conventional circumcision techniques, and there is no gold standard. Therefore, various techniques have been used with varying outcomes [5]. We report a case of CMP that underwent surgery with cosmetic satisfaction and voiding function to provide an understanding of its clinical features, management, and outcomes.

## Patient and observation

**Patient information:** a 4-year-old boy came to the urology clinic with a complaint of difficulty in micturition. His parents said that when he was undergoing micturition, the patient said he felt pain accompanied by ballooning of the preputium. The ballooning was from the accumulation of urine in the preputium. It happened since the patient was an infant, but the parents just took him for a consultation with a doctor. Also, the patient sometimes felt fever every single month but got better after taking medication. Another complaint, such as nausea and vomiting, was denied.

**Figure 1:**
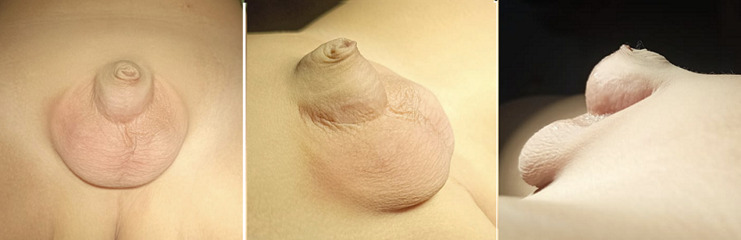
appearance of congenital megaprepuce

**Clinical findings:** on the physical examination, the vital signs revealed normal limits. Local examination of genitalia showed the penis appeared buried beneath a large amount of skin of the preputium, which was excessively redundant, and also not circumcised yet (Figure 1). During micturition, urine collects within this sac, leading to ballooning of the prepuce and urine dribbling through the small prepuce orifice. The scrotum and both testes were normal, in position and consistency. No pain on the palpation; also, there is no sign of infection, erythema, or discharge around the genital area. The laboratory test showed normal limits.

**Diagnostic assessment:** laboratory examination results did not show any abnormalities with the following description: white blood cells (WBC): 9,200/µL, neutrophils 45%, hemoglobin 12.8 g/dl, hematocrit 37%, red blood cells (RBC) 4.6 million/µL, platelets 325,000 /µL, ureum 24 mg/dL, creatinine 0.5 mg/dL, prothrombin time (PT) 12.2 seconds, and activated partial thromboplastin time (APTT) 31.5 seconds. The results of the urinalysis examination also showed normal results, with clear yellow urine, no blood or protein in the urine, all within normal limits.

**Figure 2:**
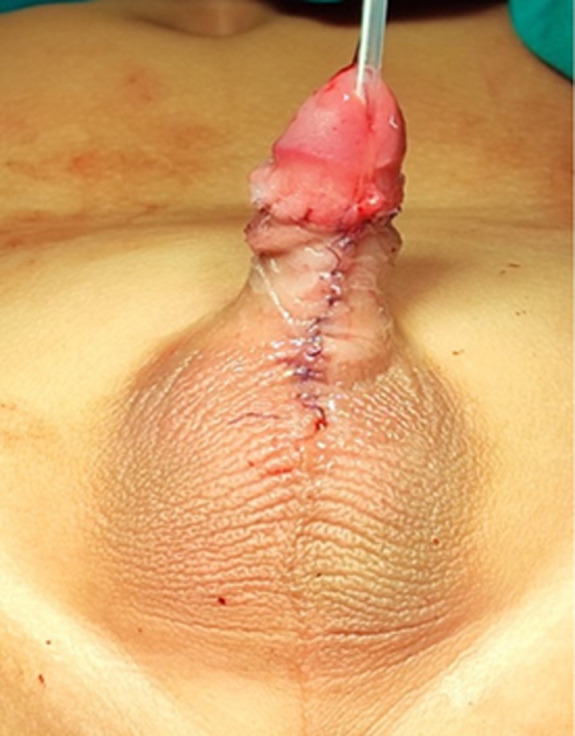
after surgical intervention, sutured in the ventral midline and circumcised

**Diagnosis:** based on the patient´s complaints and physical examination findings, we diagnosed them with congenital megaprepuce and planned to perform a surgical procedure, which included penile reconstruction and circumcision. Therapeutic interventions: The patient was planning for surgical intervention to release the glans penis with circumcision and reconstruction of the penis. The surgery was performed under general anesthesia. After disinfection around the genitalia area, the glans penis was exposed by gently opening the phimotic ring with the hemostat and 5.0 PDS suture was placed on the glans to facilitate intraoperative handling. A urinary catheter was inserted to maintain the urinary system and to prevent urethral injury. The main surgical steps are: i) penile degloving: a circumferential incision was made approximately 1cm below the coronal sulcus, followed by a longitudinal ventral cut to the penoscrotal junction to completely deglove the penis down to fascia Bucks´s; ii) removal of thickened dartos tissue: the dysplastic dartos muscle was excised extensively up to the penile base; iii) reduction of redundant inner prepuce: excess inner preputial tissue was removed and skin flaps were prepare and rotated ventrally to ensure adequate shaft coverage; iv) the penopubic and penoscrotal angles were redefined using dorsal and ventral anchoring sutures between fascia Buck´s and the dermis. The scrotum and ventral penile skin were closed in layers with absorbable sutures (Figure 2).

**Follow-up and outcome of interventions:** after the procedure, the patient was hospitalized for 2 days and maintained by antibiotics, analgesics, and wound care. Before the patient was planning for discharge, the postoperative wound showed no immediate complications such as bleeding, infection, and urinary retention. The patient and his parents are feeling satisfied with the functional voiding and cosmetic results after surgery.

**Patient perspective:** the patient's family felt greatly helped and grateful for the accurate diagnosis and appropriate treatment provided to their child. The patient and family also greatly appreciated the hard work and kindness of all the medical staff involved, which made the burden feel lighter.

**Informed consent:** written informed consent was given to the patient's family, and they agreed.

## Discussion

It is often misdiagnosed as congenital phimosis or buried penis, which may delay appropriate surgical management [2]. In the first description of CMP by O'Brien *et al*. in 1994, it was thought of as a buried penis characterized by ballooning of the excessively redundant prepuce [3]. After so many cases about CMP, it is concluded that CMP is a rare congenital anomaly of the male external genitalia characterized by redundant and abnormally distended inner preputial skin, a narrow preputial orifice, and a buried penile shaft due to abnormal development and thickening of the dartos fascia [6]. This anatomical anomaly leads to collecting urine in the preputial sac during micturition, causing ballooning of the foreskin, while anatomically normal of the meatus at the tip of glans [4]. The etiology of CMP remains unclear, but it is believed that abnormal embryologic development of the penile skin and dartos complex leads to a redundant inner prepuce and buried penis appearance [7]. Histological studies demonstrate dysplastic dartos tissue, with abnormal fascial and ligamentous attachments between the penile shaft, pubic, and scrotum. Also, the development of CMP was a failure in the separation of migrational planes in the developing male genitalia externally [8]. Classical presentation of CMP involves urine accumulation on the redundant preputial sac during voiding [3]. Some infants may show signs of discomfort, crying on micturition [4]. In our case, a 4-year-old boy complained of difficulty in micturition and had been noticed by his parents since infancy.

The ballooning of the preputium every time urinating, which subsided only after manually expressing the urine. However, the parents refuse to avoid the surgery until the child reaches school age. This may be attributed to sociocultural factors, especially in the Muslim population, which had to undergo circumcision in school age or adolescence [9]. In the few circumcised cases, most present to urology practices only if they had complications after circumcision, while some are well educated about penile conditions that cannot be circumcised in primary care settings, such as hypospadias [10]. Several reports have also described that CMP cases are complicated by urinary tract infections, likely caused by prolonged urine retention within the preputial sac or inadequate local hygiene [1]. Surgical intervention was the only way to correct the CMP and there is no evidence of spontaneous resolution [1]. There are many surgical procedures that have been described in the management of CMP, that have advantages for every technique [5]. In our case, the main purpose of surgical intervention was to resect the redundant inner prepuce and allow for normal voiding without ballooning of the prepuce to prevent urinary tract infection. After surgery, the penis was immobilized with a dressing and an indwelling catheter. The patient was subsequently discharged two days’ post-operative. The wound was shown to be well healed with a normal circumcised penis and satisfaction of cosmetic voiding function.

## Conclusion

Despite its rarity, congenital megaprepuce (CMP) needs to be differentiated from other penile anomalies such as buried penis and webbed penis. Early recognition and surgical intervention are essential to prevent complications and ensure satisfactory cosmetic outcomes for circumcised penis.
